# Frameshift mutation in SQSTM1 causes proximal myopathy with rimmed vacuoles: A case report

**DOI:** 10.3389/fneur.2023.1043136

**Published:** 2023-03-14

**Authors:** Rui Wu, Sai Shao, Ling Yin, Jianwen Deng, Shougang Guo, Lin Lu

**Affiliations:** ^1^Department of Neurology, Shandong Provincial Hospital Affiliated to Shandong First Medical University, Jinan, Shandong, China; ^2^Department of Radiology, Shandong Provincial Hospital Affiliated to Shandong First Medical University, Jinan, Shandong, China; ^3^Department of Neurology, Peking University First Hospital, Beijing, China

**Keywords:** frameshift mutation, SQSTM1, myopathy, rimmed vacuoles, multisystem proteinopathy

## Abstract

p62/Sequestosome-1 (SQSTM1) is a stress-inducible scaffold protein involved in multiple cellular processes, including apoptosis, inflammation, cell survival, and selective autophagy. SQSTM1 mutations are associated with a spectrum of multisystem proteinopathy, including Paget disease of the bone, amyotrophic lateral sclerosis, frontotemporal dementia, and distal myopathy with rimmed vacuoles (MRV). Herein, we report a new phenotype of SQSTM1-associated proteinopathy, a novel frameshift mutation in SQSTM1 causing proximal MRV. A 44-year-old Chinese patient presented with progressive limb–girdle weakness. She had asymmetric proximal limb weakness and myopathic features on electromyography. The magnetic resonance images showed fatty infiltration into muscles, predominantly in the thighs and medial gastrocnemius, sparing the tibialis anterior. Muscle histopathology revealed abnormal protein deposition, p62/SQSTM1-positive inclusions, and rimmed vacuoles. Next-generation sequencing showed a novel pathogenic SQSTM1 frameshift mutation, c.542_549delACAGCCGC (p. H181Lfs^*^66). We expanded the pathogenic genotype of SQSTM1 to include a new, related phenotype: proximal MRV. We suggest that SQSTM1 variations should be screened in cases of proximal MRV.

## Introduction

p62/Sequestosome-1 (SQSTM1) is a stress-inducible scaffold protein involved in multiple cellular processes, including apoptosis, inflammation, cell survival, and selective autophagy (1–3). SQSTM1 can serve as a scaffold for multiprotein complexes and a regulator of ubiquitinated protein turnover (1). SQSTM1 variations are associated with multisystem proteinopathies (MSPs), including Paget disease of the bone (PDB) (4), amyotrophic lateral sclerosis (ALS), frontotemporal dementia (FTD) (5), and myopathy with rimmed vacuoles (MRV) (6, 7). Coexisting SQSTM1 and *TIA1* variants are linked to MRV, presenting as late-onset distal myopathy; some cases are accompanied by cognitive impairment, dyspnea, and cardiac conduction abnormality (6, 7). Interaction between these two genes raises the possibility of digenic myopathy. Although SQSTM1/*TIA1* analysis was suggested during distal MRV investigations, isolated SQSTM1-related proximal myopathy has never been reported.

Herein, we describe a patient with proximal myofibrillar myopathy caused by a novel, heterozygous frameshift mutation in SQSTM1, expanding the clinical, pathological, and mutational spectrum of associated disorders.

This study was approved by the Ethics Committee of the Shandong Provincial Hospital, Jinan, Shandong, China, and was conducted in accordance with the Declaration of Helsinki. Written informed consent was obtained from the patient and her family members. Lower limb muscle magnetic resonance imaging (MRI) was performed using a 3.0T MR scanner (Signa Excite, Siemens, Berlin, Germany). A muscle biopsy was performed on the left biceps, and frozen muscle sections were processed using routine histochemical staining, including hematoxylin and eosin, modified Gomori trichrome, nicotinamide adenine dinucleotide dehydrogenase (NADH)–tetrazolium reductase, and cytochrome oxidase (COX) staining. Muscle sections were immunostained with specific anti-p62/SQSTM1 (Abcam) and a secondary anti-murine tetramethylrhodamine antibody. Images were acquired by confocal microscopy (Nikon A1MP). Targeted next-generation sequencing was performed using a customized Agilent SureDesign Panel Kit for neuromuscular disorders (including *VCP, hnRNPA2B1, hnRNPA1*, SQSTM1, and *MATR3*), while patient DNA was sequenced on the Illumina HiSeq sequencer (Illumina, California, USA). The pathogenicity of candidate variants was predicted by using multiple *in silico* algorithms such as Mutation Taster, FATHMM-MKL, and PROVEAN, and was classified according to American College of Medical Genetics and Genomics (ACMG) guidelines. Sanger sequencing was used to validate the filtered variants in the family members of the patient. Finally, the variants were selected due to their relationship with the disease, pattern of segregation with the disease, pattern of inheritance, allele frequency in controls, and predicted pathogenicity.

## Case description

A 44-year-old woman presented with an inability to run fast and difficulty boarding the bus and standing up from a squatting position. Born to non-consanguineous Chinese parents after an uneventful pregnancy, her motor milestone development was normal. At 38 years, she reported a progressive weakness in her lower extremities, and within 6 months, raising her arms and climbing stairs had become difficult. She was unable to stand up from a squatting position independently, even with direct assistance, until 3 months ago. The weakness was declared asymmetric and prominent in the right thigh and left arm; skeletal or muscle pain, rigidity, tremors, palpitations, and dyspnea were absent. Upon examination, she exhibited normal intelligence, and cranial nerve examinations yielded unremarkable results. A waddling gait was noticeable, while the ability to walk on toes and heels was retained. Muscle weakness was observed in the neck and proximal limbs [Medical Research Council (MRC) 3/5 in the right leg and 2/5 in the left leg]; the distal muscles were spared. Beevor's sign was positive, indicating rectus abdominis involvement. No muscular atrophy or hypertrophy was present; reflexes decreased in both the upper and lower limbs, while sensation was intact.

**Figure 1:**
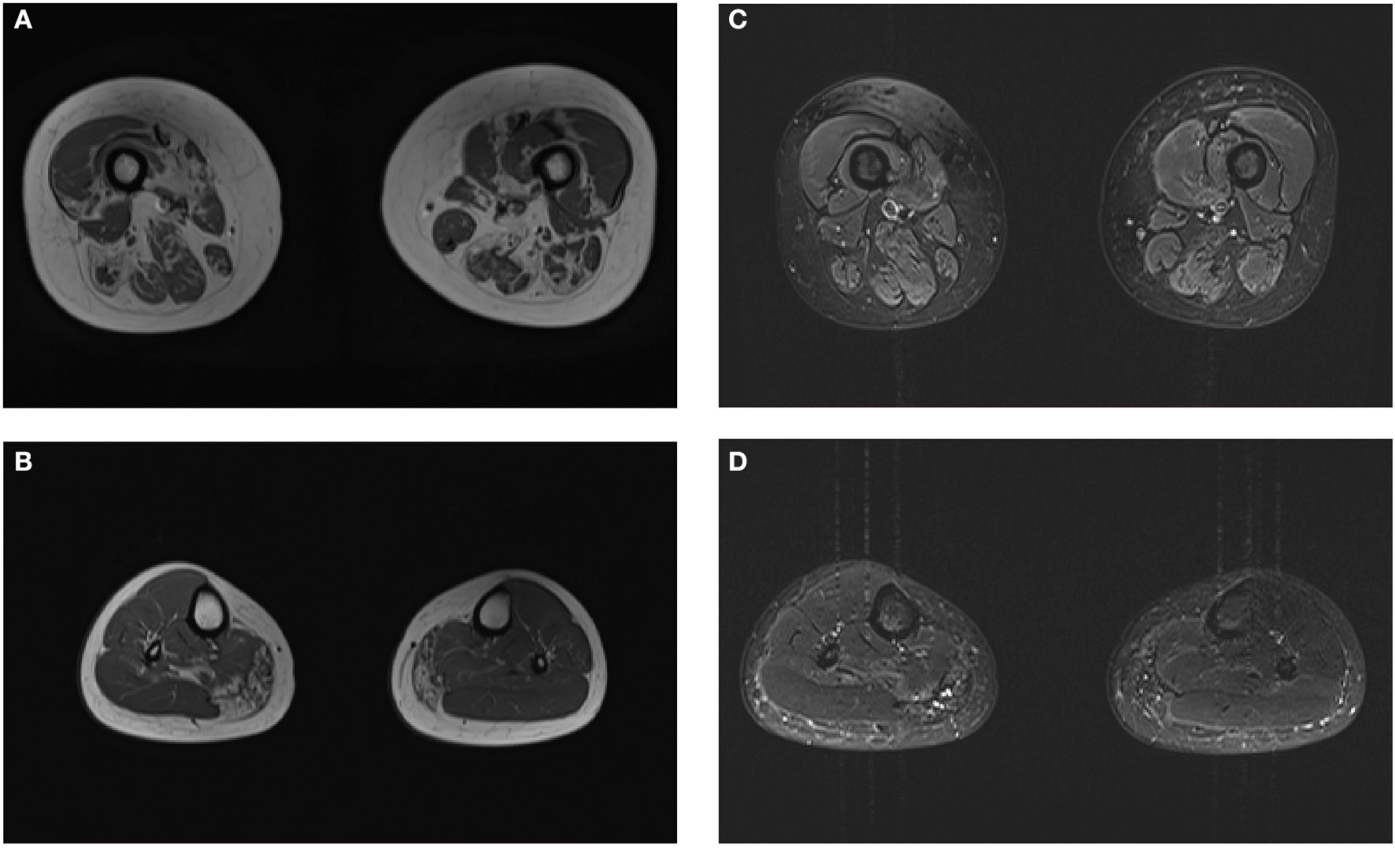
Magnetic resonance imaging (MRI) images of the patient. Muscle MRI shows the geographic distribution of muscular fatty infiltration. The predominantly affected muscles included the quadriceps femoris and posterior compartment in the thighs **(A)** and medial gastrocnemius with sparing of anterior tibialis in the lower legs **(B)**. No muscle edema was seen in the proband **(C, D)**.

Laboratory investigations indicated slightly elevated creatine kinase levels (406 U/L; normal range: 40–200 U/L). Electrocardiography and doppler echocardiography did not indicate heart conduction abnormalities or dilated cardiomyopathy. Pulmonary function tests were normal. The plain film skeletal survey failed to reveal Pagetoid lesions. MRI of the thighs indicated significant fatty replacement on the right side, which was more prominent in the posterior compartment; only the medial heads of the gastrocnemius and right soleus revealed mild degenerative changes in the lower legs (Figure 1). Electromyography studies revealed myopathic changes in the deltoid, biceps brachii, iliopsoas, and quadriceps femoris, with relative sparing of the tibialis anterior; motor and sensory nerve conduction studies revealed normal function.

**Figure 2:**
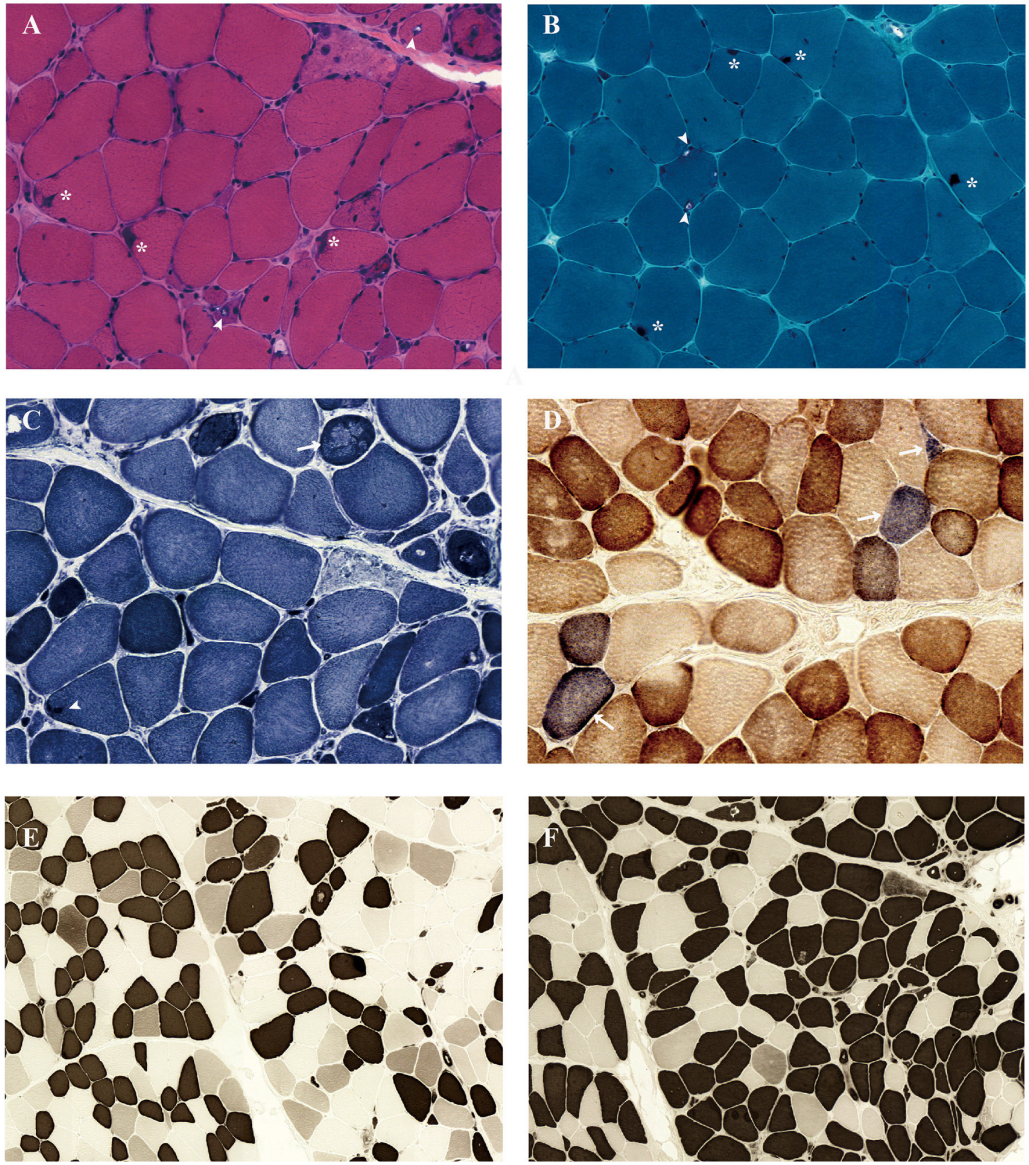
Muscle biopsy of the biceps brachii muscle demonstrated a rimmed vacuolar myopathy. Light microscopy of muscle biopsy shows size variability, excess internal nuclei, necrotizing fiber, degenerating fibers, basophilic deposition (*), and rimmed vacuoles (arrowhead) under hematoxylin and eosin staining **(A)**. Gomori trichrome staining revealed rimmed vacuoles (arrowhead) and darkly stained materials (*) **(B)**. Nicotinamide adenine dinucleotide dehydrogenase–tetrazolium reductase staining revealed decreased oxidative enzyme reactivity (arrow) and darkly stained materials (arrowhead) in some myofibers **(C)**. Combined cytochrome c oxidase (COX)/succinate dehydrogenase (SDH) stain demonstrates several COX-negative fibers (arrows) **(D)**. The fibers show the checkerboard-like distribution in ATPase 4.5 **(E)** and ATPase 9.4 stain **(F)**.

**Figure 3:**
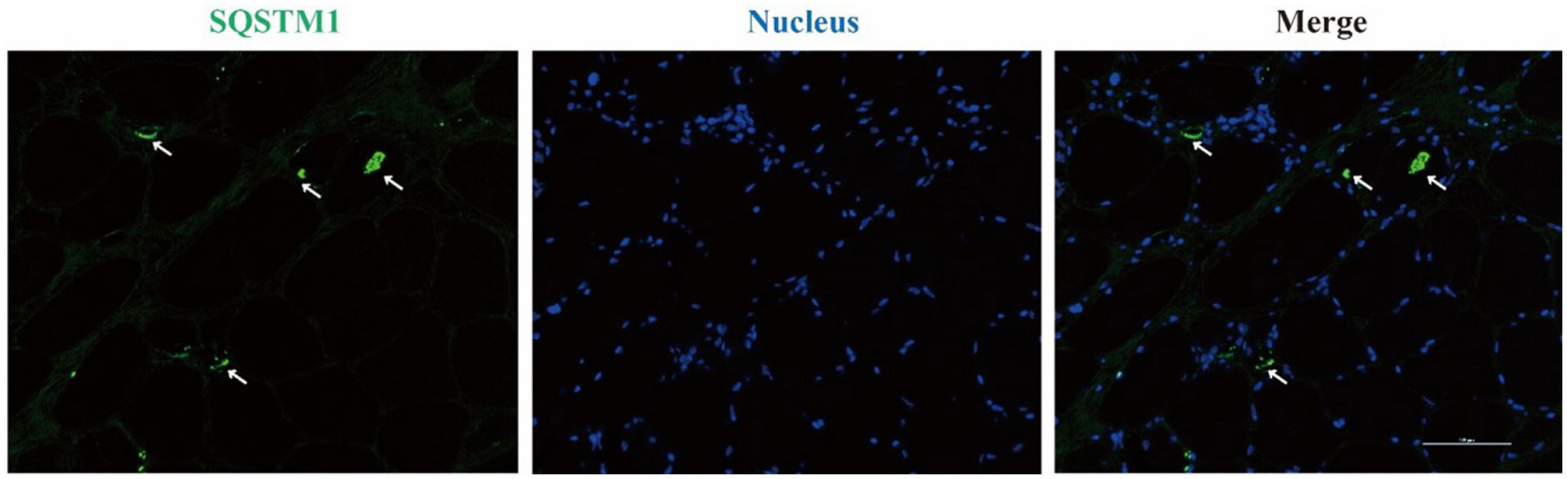
Immunofluorescence staining reveals SQSTM1-positive inclusions in rimmed vacuole marked with arrows.

Left biceps' biopsy showed an extensive presence of basophilic deposition and rimmed vacuoles (Figure 2A). Rimmed vacuoles were further confirmed using Gomori trichrome staining, and darkly stained materials were remarkable (Figure 2B); fibers focally devoid of enzyme activity were observed in NADH-stained sections (Figure 2C). Several fibers showed absent COX activity (Figure 2D). No fiber-type grouping was observed *via* ATPase 4.5 and 9.4 staining (Figures 2E, F). Immunohistochemistry revealed that the cytoplasmic inclusions were p62/SQSTM1-positive (Figure 3).

**Figure 4:**
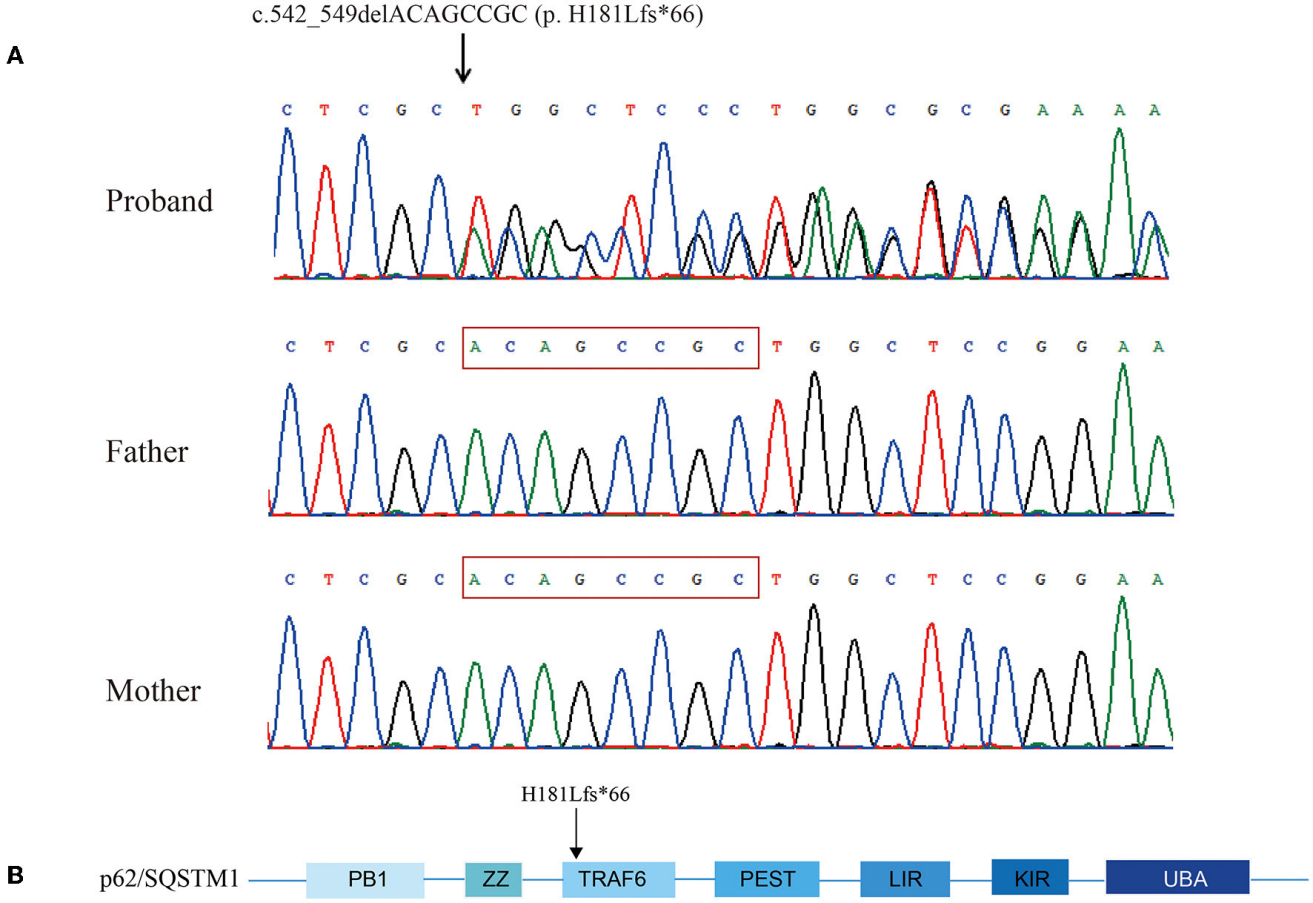
Chromatograms showing sequencing analysis of heterozygous frameshift mutation c.542_549delACAGCCGC (p. H181Lfs*66) in SQSTM1 in the proband and her parents' sequences. The red frame was the deletion part of the proband **(A)**. Schematic view of p62/SQSTM1 functional domains. PB1, Phox and Bem1; ZZ, zinc finger motif; TRAF6, TNF receptor-associated factor 6; PEST, proline, glutamic acid, serine, and threonine domain; LIR, LC3 interaction region; KIR, KEAP1 interaction region; UBA, ubiquitin association domain. The amino acid number indicates the proband's mutation **(B)**.

Targeted exome sequencing was performed for the proband and her parents. After filtering the variants, a heterozygous 8-base deletion in the SQSTM1 gene, c.542_549delACAGCCGC (NM_003900), which is localized to exon 4, was found in the proband. This new variant was predicted to induce a frameshift from amino acid 181 (p. H181Lfs^*^66), leading to the termination of transcription after 66 amino acids. Sanger sequencing confirmed the segregation of the mutation with the disease. Neither of the healthy parents was a carrier, suggesting that it was a *de novo* mutation (Figure 4A). According to the ACMG criteria, this variant was considered “pathogenic” (PVS1 + PS2). The Mutation Taster Score was 1. No other pathogenic variants were identified.

## Discussion

This is the first detailed clinical and pathological description of an autosomal-dominant proximal myopathy with rimmed vacuoles associated with a novel frameshift mutation in SQSTM1 (p. H181Lfs^*^66), and we suggest that this is a new phenotype of MSP. MSP represents a pleiotropic spectrum of rare genetic disorders, presenting with a combination of myopathy, bone disease, and neurodegeneration (8). MSP myopathy is characterized by slowly progressing weakness of the proximal, distal, cardiac, and/or respiratory muscles. Pathologically, it may overlap with myofibrillar myopathy, characterized by rimmed vacuoles, Z-disk streaming, disruption, aggregation, and mitochondrial abnormalities (6, 9, 10). In addition to SQSTM1 (6, 7), *VCP* (11), *hnRNPA2B1, hnRNPA1* (12, 13), and *MATR3* (14) have also been linked to this phenotype. Compared with *VCP* myopathy, also known as inclusion body myopathy (IBM), the most common myopathy of MSP with symmetric muscle involvement (15), the proband's myopathy was asymmetric and had p62/SQSTM1 inclusions in the myofibers. *MATR3* mutation-associated myopathy was associated with the same distal limb weakness as previous SQSTM1-related cases, while the present case revealed proximal myopathy. These diverse clinical phenotypes shared a common molecular pathogenesis—originating from the dysfunction of the ubiquitin-proteasome system and autophagy—that was responsible for protein clearance (16).

Only two previous studies illustrated myopathies associated with SQSTM1 variants (c.1165+1 G>A, c.1175C>T) (6, 7). In both, onset occurred after the fourth decade, manifesting as distal myopathy; high stepping gait and finger extensor weakness were prominent clinical features. In contrast, our case exhibited a relatively earlier onset, showing weakness in limb–girdle distribution. Waddling gait, neck flexion weakness, and positive Beevor's sign were characteristic, while distal strength was preserved; previously reported systemic involvement, such as dyspnea and arrhythmia, was not present. Nonsense mutations in SQSTM1 were also associated with frontotemporal dementia and progressive ataxia movement disorders in previous studies (17, 18). Other SQSTM1-linked phenotypes (e.g., PDB, ALS, and FTD) were excluded based on examinations, suggesting that the muscle was selectively involved.

In previous SQSTM1-related distal myopathy reports, the tibialis anterior was always predominantly involved and completely replaced by fatty tissue on muscle MRI (6, 7); in this case, it was well-preserved. Although depicting a selective pattern of muscle involvement—as with other muscular dystrophies—was difficult (19), prominent muscular fatty infiltration in the proximal limbs, with sparing of the anterior lower legs, may suggest isolated SQSTM1 non-sense mutation-associated myopathy.

As previously reported, myopathy-related SQSTM1 variants have the following pathological features: myofiber size variations, partially decreased NADH enzyme reactivity, and the presence of rimmed vacuoles, suggesting compensatory autophagic reactivity (6, 7). However, in this case, we additionally noticed the extensive presence of sub-endomysium basophilic material deposition that includes p62/SQSTM1 (20, 21), revealing severely compromised autophagy and resultant significant accumulation of abnormal protein. Previously reported type-1 fiber grouping—indicating reinnervation—was absent, implying that neurogenic mechanisms had not developed in this case. The rare presence of COX-negative fibers indicated mitochondrial affection as well, which potentially contributed to the pathogenesis.

Similar to other genes related to multisystem proteinopathy (e.g., *VCP, hnRNPA2B1*, and *hnRNPA1*) (15), pathogenic variants of SQSTM1 could lead to different diseases, such as ALS and PDB; the associated phenotypes may, thus, be affected by susceptibility alleles, rendering certain organ systems more likely to become involved. Niu et al. and Evilä et al. suggested that *TIA1* was one modifier that could prompt SQSTM1 variants to cause muscle involvement (7, 22). The combination of *TIA1* and SQSTM1 variants tend to result in myofibrillar myopathy (6, 7, 22). The *TIA1* pathogenic variant (p.N357S) was also confirmed to lead to impaired stress granule clearance and myotoxicity along with pathogenic SQSTM1 mutations experimentally (23). In the present case, however, no other pathogenic variants were identified in *TIA1* or other likely candidates for regulatory genes. The identified SQSTM1 frameshift mutation transcribed a stop codon after 247 amino acids, causing loss of the truncated p62/SQSTM1 KEAP1 interaction region (KIR) and ubiquitin-associated (UBA) domain (Figure 4B) (24, 25). The UBA domain is associated with ubiquitinated proteins, whose loss would disassociate p62/SQSTM1 and autophagic cargoes, compromising autophagy. The KIR domain is where p62/SQSTM1 docks onto KEAP1, blocking NF-E2-related factor 2 (NRF2) binding and avoiding its ubiquitylation and degradation. Therefore, the lack of a KIR domain would result in excessive NRF2 degradation, while autophagy would be less likely to be promoted, explaining why autophagic function was more severely disrupted than previously reported. We speculate that the SQSTM1 variations encoding truncated proteins could cause a dominant-negative effect and are less likely to be modified by other genes; further research is required to verify this hypothesis.

In conclusion, we expanded the pathogenic genotype of SQSTM1 and related phenotypes to include proximal MRV. The characteristic histopathology reflected the underlying pathogenesis. We suggest proximal myopathy to be another subtype of SQSTM1-associated MRV.

## Data availability statement

The datasets presented in this article are not readily available because of ethical and privacy restrictions. Requests to access the datasets should be directed to the corresponding author/s.

## Ethics statement

The studies involving human participants were reviewed and approved by the Ethics Committee of Shandong Provincial Hospital, Jinan, Shandong, China. The patients/participants provided their written informed consent to participate in this study. Written informed consent was obtained from the individual for the publication of any potentially identifiable images or data included in this article.

## Author contributions

RW: conceptualization, writing—original draft, review and editing, project administration, and funding acquisition. SS: performing muscle MRI. LY and JD: performing immunohistochemistry of muscle biopsy. SG: performing EMG. SG and LL: resources and review and editing. All authors contributed to the article and approved the submitted version.
